# Genetic Analysis, Population Structure, and Characterisation of Multidrug-Resistant *Klebsiella pneumoniae* from the Al-Hofuf Region of Saudi Arabia

**DOI:** 10.3390/pathogens10091097

**Published:** 2021-08-28

**Authors:** Lorina I. Badger-Emeka, Abdulrahman A. Al-Sultan, Marie Fe F. Bohol, Mashael R. Al-Anazi, Ahmed A. Al-Qahtani

**Affiliations:** 1 Department of Biomedical Sciences, College of Medicine, King Faisal University, Al-Ahsa 31982, Saudi Arabia; 2 College of Applied Medical Sciences, King Faisal University, Al-Ahsa 31982, Saudi Arabia; aalsultan@kfu.edu.sa; 3 Department of Infection and Immunity, Research Centre, King Faisal Specialist Hospital and Research Centre, Riyadh 11211, Saudi Arabia; mbohol@kfshrc.edu.sa (M.F.F.B.); masheal2008@hotmail.com (M.R.A.-A.); aqahtani@kfshrc.edu.sa (A.A.A.-Q.); 4 Department of Microbiology and Immunology, College of Medicine, Alfaisal University, Riyadh 11211, Saudi Arabia

**Keywords:** *Klebsiella pneumoniae*, antimicrobial, multidrug, resistance, clonal, extended-beta lactamase, genes

## Abstract

Multidrug-resistant *Klebsiella pneumoniae* (MDR-KP) is a major public health problem that is globally associated with disease outbreaks and high mortality rates. As the world seeks solutions to such pathogens, global and regional surveillance is required. The aim of the present study was to examine the antimicrobial susceptibility pattern and clonal relatedness of *Klebsiella pneumoniae* isolates collected for a period of three years through pulse field gel electrophoresis (PFGE). Isolate IDs, antimicrobial assays, ESBL-production, and minimum inhibitory concentrations (MICs) were examined with the Vitek 2 Compact Automated System. IDs were confirmed by 16S rRNA gene sequencing, with the resulting sequences being deposited in NCBI databases. DNA was extracted and resistance genes were detected by PCR amplification with appropriate primers. Isolates were extensive (31%) and multidrug-resistant (65%). Pulsotype clusters grouped the isolates into 22 band profiles that showed no specific pattern with phenotypes. Of the isolates, 98% were ESBL-KP, 69% were carbapenem-resistant Enterobacteriaceae (CRE) strains, and 72.5% comprised the carriage of two MBLs (SIM and IMP). Integrons (ISAba1, ISAba2, and IS18) were detected in 69% of the MDR-KP. Additionally, OXA-23 was detected in 67% of the isolates. This study therefore demonstrates clonal diversity among clinical *K. pneumoniae*, confirming that this bacterium has access to an enormous pool of genes that confer high resistance-developing potential.

## 1. Introduction

*Klebsiella pneumoniae* (*K. pneumoniae*), an opportunistic pathogen, was first described by Carl Friedlander in 1882 [1]. It is an encapsulated Gram-negative bacterium belonging to the Enterobacteriaceae family, and it is found residing in the gastrointestinal and oral cavities of humans. *K. pneumoniae* shows diversity in the colonisation of other parts of the human body such as the skin and respiratory tract [2], and it has been associated with a wide range of hospital- and community-acquired infections. The majority of infections by this opportunistic pathogen are associated with the elderly, the immunocompromised, and neonates, where urinary tract infections, wound and soft tissue infections, and pneumonia are the most commonly reported. Intensive care unit (ICU)-related infections, sepsis, and infections of surgical site are additionally reported [3,4,5,6,7]. *K*. *pneumoniae* is generally listed as one of the most frequent causes for hospital-acquired infections (HAIs) [8], with organisations such as the European Union (EU), World Health Organisation (WHO), and the Centres for Disease Control and Prevention (CDC) recognising the bacterium to be a significant threat to global public health [9,10]. This is the result of difficulties in treating strains of *K. pneumoniae* that exhibit multidrug resistance (MDR) to available antibiotics. Additionally, disturbing is the heavy disease burden attributed to this bacterium in critically ill patients [11], thus leading to poor treatment outcomes [12,13,14]. Moreover, *K. pneumonia* infections are associated with extended hospital stays, along with subsequent high mortality rates resulting from isolates that are extremely resistant (XDR) against most or sometimes all antibiotics [15]. Infections caused by *K. pneumoniae* are therefore receiving continual attention in surveillance with the aim of gaining adequate control of such bacterial superbugs. In 2013, the CDC attributed about 80% of 9000 Enterobacteriaceae infections that were resistant to carbapenems to be those of *K. pneumonia* [1]. The bacterium has continued to receive global attention since its first discovery, probably due to a wide range of factors that lead to both infections and antimicrobial resistance [16,17]. In Saudi Arabia, research on *K. pneumoniae* infections has been consistent [18,19,20,21]. Most studies have been retrospective, covering in vitro infection patterns and their trends of susceptibility to antimicrobial agents used in the treatment of patients. Concerns have generally been expressed regarding the high resistance of HAIs, particularly with isolates that are extended-spectrum beta lactamase (ESBL)-producing, of which 300 variants have been reported [22,23]. In a recent report, the first *K. pneumoniae* carbapenemase-producing (KPC) Enterobacteria isolated from a urine specimen of a patient with travel history outside the Kingdom of Saudi Arabia was reported [20]. Though such strains had been reported in other parts of the world such as the United Kingdom (UK), United States of America (USA), Greece, and Italy [24], this was a rare occurrence in Saudi Arabia. Such reports demonstrate how vital the continuous surveillance of the antimicrobial resistance of bacterial isolates is, as this could be useful in guiding clinicians in the implementation of empirical treatment, as previously reported [19]. With the rise in antimicrobial resistance and ICU hospitalisation being a high-risk factor in HAIs, there is need for the continuation of global and regional surveillance to appropriately mitigate *K. pneumoniae* nosocomial infections. It has been suggested that the efficient global and regional genotyping of *K. pneumoniae* isolates is important for such surveillance. It has been suggested that this could be an effective tool for monitoring and controlling the spread of epidemic-associated clones [25].

Pulsed field gel electrophoresis (PFGE) was proposed to be the gold standard for bacterial genotyping due to the ability of this typing method to cut DNA into small fragments that can aid in the recognition of the source and possible species associated with hospital outbreaks [25]; the author of that report hypothesised that clinical *K. pneumoniae* isolates in this region of study might exhibit genetic-phenotypical diversity. The authors of the present research considered the antimicrobial susceptibility pattern and clonal relatedness of the *Klebsiella pneumoniae* isolates collected for a period of three years in the region of study by PFGE. Resistance determinants and the presence of genetic elements such as IS*Aba*1, IS*Aba*2, IS*Aba*3, and IS18 associated with *K. pneumoniae* were ascertained. Isolate IDs were confirmed by 16S rRNA sequencing. The research was conducted with the intention of bridging the gap in literature regarding the genetic relatedness of *K. pneumoniae* isolates in the Al-Hofuf region of Saudi Arabia, ultimately for the purposes of the required global and regional surveillance for monitoring and possibly controlling MDR bacteria.

## 2. Methods

### 2.1. Bacterial Isolates

A total of seventy-eight non-repetitive clinical *Klebsiella pneumoniae* isolates collected over a period of 3 years were randomly selected from a microbial bank for the study. These isolates had been stored at a temperature of –80 °C in the Microbank at the Department of Medical Microbiology. They were plated out on MacConkey agar and were aerobically cultured for 24 h at a temperature of 37 °C. The ID and AST cards of the Vitek 2 Compact Automated System (BioMerieux, Marcy L’Etoile, France) were used for bacterial identification and antimicrobial susceptibility tests according to the guidelines of the manufacturers. The GN AST antimicrobial cards comprised the following antibiotics: ampicillin/sulbactam (Ams), amoxicillin/clavulanic acid (Aug), amoxicillin (Aml), piperacillin/tazobactam (Ptz), ceftazidime (Caz), cefepime (Pime), cephalotin (Kf), cefoxitin (Ctt), ceftriaxone (Cro), cefuroxime (Cxm), aztreonam (Azt), ertapenem (Etp), imipenem (Imp), meropenem (Mer), amikacin (Amk), gentamicin (Gn), tobramycin (To), ciprofloxacin (Cp), minocycline (Min), norfloxacin (Nor), levofloxacin (Levo), nitrofurantoin (Fd), colistin (Cst), tigecycline (Tig), and trimethoprim/sulfamethoxazole (Ts). The minimum inhibitory concentrations and the ESBL production of the isolates were determined with the Vitek 2 Compact Automated System (BioMerieux, Marcy L’Etoile, France). Each of the *K. pneumoniae* isolates resistant to ceftriaxone and cefotaxime were phenotypically characterized by a modified double disc synergy test (MDDST) according to CLSI [26] recommendations. Tests separately constituted ceftazidime and cefotaxime before combining either with clavulanic acid. The interpretation of the results was conducted as previously described [26,27].

### 2.2. The Extraction of Genomic DNA and PFGE Protocol for Klebsiella pneumoniae

The genomic DNA extraction of the *K. pneumoniae* isolates was conducted using a Qiagen DNA extraction kit following the guidelines of the manufacturers. For PCR amplification, we used a total volume of 50 μL constituting 25 μL of a master mixture, 2 μL of each of the universal primers 8F (5′AGAGTTTGATCCTGGCTCAG3′) and 1942R (5′GGTTACCTTGTTACGACTT3′) as well as a 100 ng DNA template. The PCR conditions were: 5 min of denaturation at 95 °C and 30 cycles at 94 °C for 1 min; the annealing temperature for the specific primers was set to 53.8 °C for 45 s, and the extension was set to 72 °C for 1 min. The final extension took place for 5 min at a temperature of 72 °C. The resultant PCR products were stained with 10 mg/mL of ethidium bromide and were resolved by electrophoresis on 1% agarose gel.

The clonal relatedness of the 52 randomly selected *K. pneumoniae* isolates was explored by PFGE using the standardised PulseNet One-Day PFGE protocol [28,29,30,31]. For each isolate, a single bacteria colony was inoculated into 3 mL of soy broth (Sigma T8907) and was incubated at 37 °C for 24 h in a shaker incubator. Cells that had undergone overnight growth were harvested through centrifugation at 3000 rpm for 10 min, and the resultant supernatant was discarded.

Harvested cells were re-suspended in an SE buffer composed of 25 mM EDTA (pH 8.0) and 75 mM NaCl (pH 8.0), with the optical density (OD) of the bacterial cells adjusted to 1.4 at 610 nm using a spectrophotometer under ice conditions. To embed agarose with bacteria, equal volumes of a bacteria suspension (0.7 mL) and a 0.7 mL 2% low-melt point (LMP) agarose (Invitrogen) were mixed together. The resultant mixture was pipetted into a 300 µL reusable plug mould, and the gel plugs were allowed to solidify at 4 °C for 30 min. Plugs were then introduced into 15 mL tubes containing 1 mL of a lysis buffer onto which 10 µL of proteinase K (Thermo Fisher) were added. This set-up was incubated overnight on a water bath at a temperature of 55 °C. The washing step was as previously reported [32], and the plugs were directly treated for restriction digestion. A total of fifty units of XbaI restriction enzyme (New England Biolabs) were used for genomic DNA digestion according to the recommendations of the manufacturers, while the separation of the restriction fragments was achieved with a CHEF-DR III System (Bio-Rad Laboratories, Hercules, CA, USA).

The CHEF-DR III System was also used for electrophoresis under the following electrophoresis conditions: volts of 6 V/cm at a 120-degree angle for a duration of 22 h at a temperature of 14 °C. The pulse time was 2.16 s of the initial switch time and 54.17 s of final switch time. Gels were stained with ethidium bromide, and the bands were visualised with an ultraviolet illuminator. A 50–1000 kb DNA Lambda ladder (New England Biolabs) was used as the size of the molecular marker. Images were captured on a Gel Doc 2000 system (Bio-Rad laboratories).

### 2.3. Analysis of PFGE

*Klebsiella pneumoniae* PFGE profiles were computationally analysed with BioNumerics, version 7.5 (Applied Maths, Sint-Martens-Latem, Belgium). The unweighted pair-group with arithmetic average (UPGMA) method was used to generate the dendrogram and comparisons of band patterns with a 4% Dice similarity coefficient and 2% tolerance. The interpretation of the band patterns was conducted as previously recommended [33]. Indistinguishable patterns were given same name types, and related patterns were allotted same letters in their typed names.

### 2.4. 16s rRNA Sequencing of the Isolates and Analysis

Amplified DNA products were purified with the QIAquick PCR Purification Kit (Qiagen, USA). Bidirectional sequencing was conducted using the same forward and reverse primers for PCR amplification with Big Dye version 3.1 kit (Applied Bio-Systems) on an ABI-PRISM 3730 DNE Sequencer. Chromas (Version 2.01) was used for the correction of ambiguous sequences, and sequences were assembled with Bio-Edit version 7.0. The NCBI BLASTn program (http://www.ncbi.nih.gov/BLASTprogram, accessed on 31 March 2021) was used to search for the sequence homologs of potential isolates for species identification. The obtained nucleotide sequences were submitted to GenBank for reference.

### 2.5. Detection of Carriage of Antimicrobial Resistance Genes by Multiplex PCR

Standard multiplex PCR using the primers listed in Table 1 was used to determine carriage of some resistant genes from each of the *K. pneumoniae* isolates. The Qiagen Multiplex PCR Kit (for 100 × 50 µL multiplex PCR reactions: 2 × Qiagen Multiplex PCR Master Mix (providing a final concentration of 3 mM MgCl2, 3 × 0.85 mL), 5 × Q-Solution (1 × 2.0 mL), RNase-Free Water (2 × 1.7 mL)) was used according to the manufacturer’s guidelines. The multiplex reaction mix was added to the 1 µL extracted DNA templates of each of the isolates, with a final reaction volume of 50 µL used for the assay. Cycling conditions were also as recommended by the manufacturers: Aa initial HotStar Tag DNA polymerase heat activation occurred at 95 °C for 15 min, with 3 cycling steps comprising 30 s of denaturation at 94 °C, and annealing for 90 s at 63 °C followed by 90 s of extension at 72 °C. There were 45 circles in total, with a 10 min final extension at a temperature of 72 °C (www.qiagen.com/HB-0453, accessed on 19 June 2021). We used 2% agarose gel electrophoresis to analyse the amplified PCR products, which were then stained with ethidium bromide and visualised with a UV transilluminator.

### 2.6. Statistical Analysis

Data were analysed with GraphPad Prism, version 9. 2.0 (283). Antimicrobial susceptibility is presented as percentages. We employed two sample *t*-test between percentages via Statistic Calculator (StatPac version 4) to determine significant differences in the carriage of resistance determinant genes among the *K. pneumoniae* isolates taken at *p* < 0.05.

## 3. Results

### 3.1. Antimicrobial Susceptibility Pattern of the Isolates

**Figure 1:**
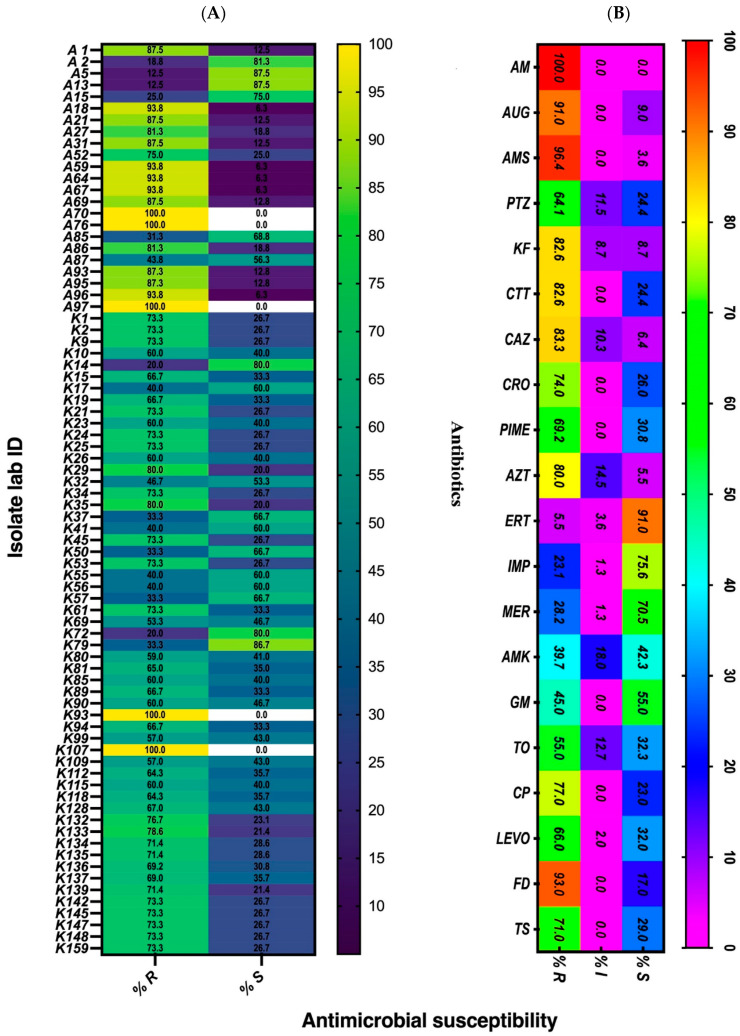
Antimicrobial susceptibility of 78 isolates of *K. pneumoniae* (**A**) and heatmap of percentage of antimicrobial susceptibility to the antibiotics used in treatment (**B**). Ampicillin/sulbactam (AMS), amoxicillin/clavulanic acid (Aug), amoxicillin (Am), piperacillin/tazobactam (Ptz), ceftazidime (Caz), cefepime (Pime), cephalotin (Kf), cefoxitin (Ctt), ceftriaxone (Cro), cefuroxime (Cxm), aztreonam (Azt), ertapenem (Etp), imipenem (Imp), meropenem (Mer), amikacin (Amk), gentamicin (Gn), tobramycin (To), ciprofloxacin (Cp), levofloxacin (Levo), nitrofurantoin (Fd), and trimethoprim/sulfamethoxazole (Ts).

Our results showed the *K. pneumoniae* isolates AB70, AB76, AB97, and AB93 to be extensively drug-resistant (XDR), as they were found to be non-susceptible to more than one agent in categories of tested antimicrobial agents exhibiting 100% resistance (Figure 1A). Though the isolates were clinical samples, only AB97 was from an ICU patient (Table 2). The other XDR isolates were AB59, AB64, AB67, AB69, AB95, AB96, and K107. Of these, only one isolate (K107) was collected two years prior to the others in the group. The majority (65%) of the remaining isolates were either multidrug-resistant (MDR) and were non-susceptible to more than one agent in three categories of antibiotics or showed moderate susceptibility (4%). The results in Table 2 show which of the antimicrobial categories each of the *K. pneumoniae* isolate was grouped into. There was an overall high resistance against antimicrobials used in the treatment of *K. pneumoniae* infections, but some strains were susceptible to antibiotics. The susceptibility pattern against 20 of the tested antibiotics is shown in Figure 1B. All isolates were resistant to amoxicillin (Am), and resistance was high for ampicillin/sulbactam (96.4%) and amoxicillin/clavulanic acid (91%) as well as others such as cefoxitin (82.6%), ceftazidime (83.3%), and aztreonam (80%). Antimicrobial sensitivity was high among the carbapenems (ertapenem, meropenem, and imipenem), though some of the isolates were found to be resistant to this group of antibiotics. However, resistance in this group was varied, as more isolates (23% and 28%) were resistant to imipenem and meropenem, respectively, compared to ertapenem (5.5%) (Figure 1B).

A total of fifty-two *K. pneumoniae* isolates were used for the PFGE assay. In Table 2, the laboratory code, source of microbial isolation, description, and exhibited pattern of drug resistance for each of the isolates is shown. Significantly, 98% of the isolates were ESBL (ESBL-KP) producers, and one isolate (K1) was found to be resistant to colistin by the Vitek 2 Compact Automated System (BioMerieux, Marcy L’Etoile, France), with an MIC of ≥16 (https://www.nih.org.pk/wp-content/uploads/2021/02/CLSI-2020.pdf, accessed on 1 of August 2012) [40].

### 3.2. Genotyping of Klebsiella pneumoniae Isolates by PFGE

**Figure 2:**
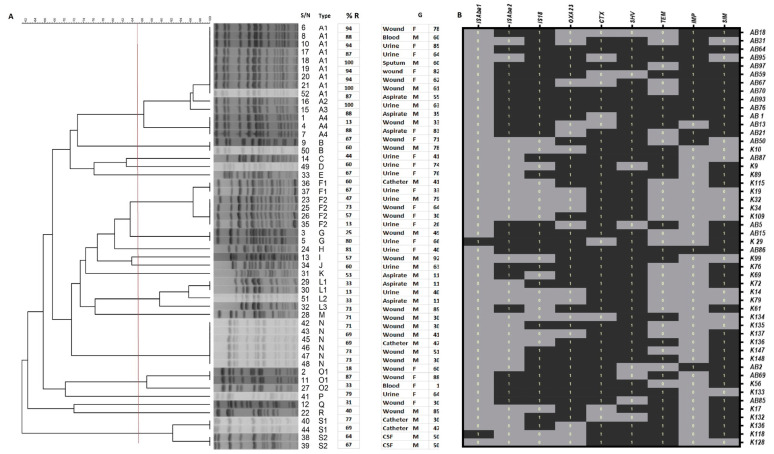
Dendrogram of pulsed field gel electrophoresis of 52 clinical isolates of Klebsiella pneumoniae (**A**). An 85% cut-off level is indicated with a red line on the dendrogram, while the clusters are grouped as letters A–S with sub-clusters. (**B**) A heatmap showing the distribution of associated resistance-determinant genes. Carriage of insertion sequence, ESBL variants (TEM, SHV, and CTX), and MBLs (SIM and IMP) are shown. Detected (1); not detected (0).

The detected PFGE patterns of the isolates displayed a genetic similarity coefficient that ranged between 62 and 100% (Figure 2A). Based on the results of PFGE, 22 band profiles were shown by the 52 isolates. Cluster A, with 14 isolates, was the largest, and the other clusters (F and N) had 6 members each. The L and S clusters each had four members of K. pneumoniae isolates, while there were two members each in clusters B and G. the remaining were singletons (E, H, I, J, K, H, J, K, P, Q, and R), with one isolate each. Cluster A consisted of four K. pneumoniae strains (A1, A2, A3, and A4). Sub-cluster A1 had nine isolates of clonally indistinguishable members, and the A2 and A3 sub-clusters (each with one isolate of K. pneumoniae) were closely related to A1. The remaining four members in the A4 sub-cluster had the possibility of being clonally related to A1 (Figure 2A). Additionally, the F cluster had six members in two sub-clusters. F1 had two clonally indistinguishable members, and F2 had four members that were clonally related to F1. The clusters with one isolate each (B, C, D, and E) were all clonally indistinguishable. There were the six members in N cluster that were additionally undistinguishable as well as those of the remaining clusters (H. I, J, R, P, Q, and R).

### 3.3. Associated Resistance Genes by Multiplex PCR

The genotype characteristic distribution of each *K. pneumoniae* isolate is shown in Figure 2B. There were multi-carriage-of-resistance-determinant genes among the isolates (Figure 2B), with the least number detected in the isolates being between two (K14, K79, and K134) and three (K10, K34, K109, and K128). IS*Aba1* was the least identified integron among the other identified insertion sequences (IS*Aba*2 and *IS*18). The highest numbers of detected genes by PCR were seen in isolates AB93 and AB86, and IS*Aba1* was not detected in either (Figure 2B). The single carriage of insertion sequence (*IS*) was more statistically significant for IS18 (21.6%) than for IS*Aba*1 and IS*Aba*2 (2% each). Additionally, IS*Aba2* and IS*18* exhibited more co-existence among the isolates (39%), but (31.4%) no *IS* was detected in some of the isolates (Table 3). However, differences between them and the number of isolates in which they were seen (69%) were statistically significant (Table 3).

There were two OXA carbapenemase-encoding genes (OXA-23 and OXA-40) that were probed, but only one (OXA-23) was detected in 67% of the isolates. There was significant difference between the OXA-23-positive and -negative samples (*p* < 0.001). A total of two MBLs (SIM and IMP) were detected in 72.5% of the isolates; SIM was detected significantly more (43%) than IMP (6%). Generally, the overall carriage (72.5%) was more significant (*p* < 0.001) than no carriage of either SIM or IMP. Among the ESBLs, TEM, SHV, and CTX were detected in the *K. pneumoniae* isolates. TEM was the least detected as single a carriage (2%) or as a co-existence with either SHV (9.8%) or CTX (7.8%). Co-existing carriages of SHV and CTX by the isolates was more significant (*p* < 0.01) (43%) than those of all three (TEM, SHV, and CTX) (27%). Only in one isolate (2%) were no ESBL genes detected (Table 3).

### 3.4. K. pneumoniae 16S rRNA Molecular Genotyping

A total of thirty *Klebsiella pneumoniae* 16S rRNA genes for the isolates from the region of the present study were sequenced, with the resultant nucleotides deposited as partial sequences in the NCBI database with the following accession numbers: MW832545, MW832549, MW832550, MW832551, MW832557, MW832719, MW832754, MW832830, MW832844, MW832864, MW832865, MW832869, MW834322, MW834429, MW834593, MW834563, MW834636, MW834640, MW834874, MW835156, MW835173, MW835179, MW835227, MW835343, MW835344, MW843597, MW843606, MW843607, MW843609, and MW843630. The web links to each of them are provided in Supplementary Table S1.

## 4. Discussion

*Klebsiella pneumoniae* is an opportunistic pathogen, and it was shown in this study to be responsible for a wide range of infections in hospitalised patients as well as in those attending outpatient departments (OPDs). The majority of the isolates were found to be from wounds, aspirate urine, and catheter tips that were mostly collected from male patients. These findings are similar to those of other researchers [41,42,43]. Most of the isolates in this study were from hospitalised, immunocompromised patients, which shows the role this opportunistic pathogen plays in HAIs, a view also expressed by others [42,44,45].

Through 16S rRNA gene sequencing, all isolates were confirmed to be of *K. pneumoniae.* However, diversity was seen in the antimicrobial susceptibility pattern, as isolates could be grouped into susceptible strain (SS), multidrug-resistant (MDR-KP), and XDR-KP groups. We observed resistance against quinolones (ciprofloxacin and levofloxacin), various groups of cephalosporins (ceftazidime, cefepime, cefotaxime, and cefoxitin), tetracyclines (minocycline), and (to some degree) carbapenems (imipenem and meropenem) and aminoglycosides (gentamicin, tobramycin, and amikacin). These results further highlight the global public health threat resulting from MDR bacterial isolates. Similar but varying findings had been reported by others in the region of this investigation [42,43] as well as in other regions of the world [46,47]. The majority of the isolates in this investigation were ESBL (ESBL-KP) strains, a reasonable number of which were carbapenem-resistant Enterobacteriaceae (CRE), and there was 100% resistance to amoxicillin. Complete resistance to ampicillin had been previously reported [41,43,48], but *K. pneumoniae* has also been reported to be intrinsically resistant to the antibiotic (ampicillin) as a result of the carriage in the genome core of the SHV beta-lactamase [2]. This drug is therefore not the natural drug of choice in the treatment of *K. pneumoniae* infections, and its reported resistance was not unexpected. Nonetheless, both of them are broad spectrum and not suitable for the management of *K. pneumoniae* [49]. The isolates in this investigation were also highly resistant to ampicillin/sulbactam (AMS), amoxicillin/clavulanic acid (Aug), piperacillin/tazobactam (Ptz), ceftazidime (Caz), cefepime (Pime), cephalotin (Kf), cefoxitin (Ctt), ceftriaxone (Cro), and cefuroxime (Cxm). These antibiotics have also been reported to be amongst the least effective against *K. pneumoniae* isolates [25,42]. In Saudi Arabia, highly resistant profiles to penicillins [43,50], cephalosporins, carbapenems, and nitrofurantoin have been described. However, the least common resistance in this study was found to be against carbapenems [48], aminoglycosides [51], and quinolones [50]. The isolates in this study were highly susceptible to carbapenems and tigecycline, both of which had also been previously reported [42] as favorable in the management of *K. pneumoniae* infections. Generally, the management of such infections is reported to rely on colistin and tigecycline, as resistance to these antibiotics are rare upon the initial isolation of the bacterium [52]. The fact that only one isolate (KP1) was resistant to colistin in this investigation could mean that resistance had arisen due to mutation during treatment, while the remaining isolates could have been of first isolation [2]. Nonetheless, while resistance was 23% and 28% for imipenem and meropenem, respectively, in this investigation, higher levels of resistance (55%) to both antimicrobials have been reported in other regions of the world [25,53,54,55]. Differences in reports suggest that these variations could be due to the clonal differences in those reported studies. The isolates in the investigation of Hashemizadeh et al. [25] were New Delhi metallo-beta-lactamase (NDM-1) strains, with no detection of KPC, VIM, or IMP genes. However, the isolates in the present study were not strains of NDM-1, but a significant number of SIM and IMP genes (78%) was detected by PCR. These differences reflect the previous description of *K. pneumoniae* populations as “an open pan genome” with access to an enormous pool of genes [56], all of which contribute to clonal diversity.

In this investigation, the majority of the isolates were found to be polyclonal based on their exhibited antimicrobial resistance gene carriages. The 98% ESBL-KP in this study was high, with only 2% in which TEM, SHV, and CTX-M were not detected. This trend is worth considering because these genes are capable of being transferred on mobile genetic elements (MGEs) in facilitating a horizontal spread of genetic resistance determinants to patients, as previously described [57]. Polyclonal ESBL-KP was also reported [58] in a non-outbreak hospital environment and was attributed to possible importation into hospital settings. Generally, the numbers of ESBL-producing bacterial pathogens have increased in the past few decades, with geographical and regional disparity [59]. Thus, K. *pneumoniae* isolates such as those in this report pose immense challenges to clinicians in the management of multidrug-resistant infections by these isolates. Additionally, of concern to clinicians is the dissemination of resistance determinant genes by MGEs, as different resistant genes are said to be associated with specific MGEs. ESBLs is plasmid-mediated and is frequently found in strains of *K. pneumoniae*, and integrons being associated with *K. pneumoniae* suggest a wide spread by MGEs. The role MGEs play in the mobility of resistant genes has contributed to strains that sometimes affect the chromosomal composition of bacteria [60,61,62], which has created a wide genomic diversity in *K. pneumoniae*; such diversity was observed in this study. A total of four integrons (IS*Aba1*, IS*ABa*2, IS*Aba*3, and IS18) were examined and were found to be associated with the spread of carbapenemase genes [63]. Of these, IS*Aba1* was the least detected, which was in contrast to other findings in which they were reported in a higher percentage [64], this could have had a number of contributory factors such as the used primers and differences in PCR amplification processes.

However, IS*Aba2* and IS18 were detected by PCR in 69% of our isolates. With regard to *K. pneumoniae* the bacterium has been reported to show several different elements of IS that could be located in a strain, all off which contribute to clonal variations in MDR-KP [65]. Though different IS elements have been reported to contribute to the mobilization and expression of antimicrobial resistance genes [66,67,68]. The role of IS18 in the dissemination of such genes in *K. pneumoniae* might need to be investigated, as there have yet to be any reports on this subject within our region of study. Thus, the 69% IS rate seen here points to these genes being antimicrobial-resistant promoters associated with the MDR-KP in this region, as suggested by the K. *pneumoniae* strains showing genotype pattern diversity in this study. These strains were seen to be genetically diverse, with phenotypic differences in their susceptibility to antimicrobials. As in our study, findings regarding genetic diversity in isolates have varied among researchers [69,70,71,72]. Additionally, the PFGE results in the present study revealed no specific pattern of association between pulsotype cluster analyses and the resistant phenotypes of the isolates, which may be a reflection of the genomic characteristics of this Gram-negative opportunistic pathogen in which the plasmid genome and chromosomal genes are large (as previously documented [59]). It is possible that MDR could be more plasmid-based than chromosomal. The general geographical and regional differences globally associated with MDR bacteria are worth considering. However, our findings are consistent with those of Akya et al. [69], who reported no specific association between antimicrobial-resistant phenotypes and PFGE cluster analysis. The first detection of OXA-23 in our isolates is noteworthy; there have been no literature reports regarding this type of finding, which will need to be studied further.

## 5. Conclusions

In summary, the present study used PFGE analysis to confirm that *K. pneumoniae* is “an open pan genome” that is associated with clonal diversity, as the majority of its genes are polyclonal. Additionally, our study revealed multi-carriage-of-resistance-determinant genes among the investigated isolates. A single carriage of insertion sequence (*IS*) was more statistically significant for IS18 than IS*Aba*1 and IS*Aba*2. The number of MBLs detected was high, thus indicating high antimicrobial resistance. A significant number (98%) of the isolates in this investigation were ESBL-KP strains, with marked carbapenem-resistant Enterobacteriaceae (CRE) strains. All of the findings suggest a high phenotypic antimicrobial and genomic diversity. In addition, we reported the presence of the OXA-23 gene for the first time, which will need to be studied further.

## Figures and Tables

**Table 1:** List of primers, oligonucleotide sequences, and sizes (bp) used in the study.

Name of Primer	Nucleotide Sequence	Amplicon Size (bp)	Reference
OXA 23-F	GATCGGATTGGAGAACCAGA	501	[34]
OXA 23-R	ATTCTTGACCGCATTTCCAT		
SIM-F	TACAAGGGATTCGGCATCG	570	[35]
SIM-R	TAATGGCCTGTTCCCATGTG		
IMP-F	CATGGTTTGGTGGTTCTTGT	616	[36]
IMP-R	ATAATTTGGCGGACTTTGGC		
ISAba1-F	GTGCTTTGCGCTCATCATGC	430	[37]
ISAba1-R	CATGTAAACCAATGCTCACC		
ISAba2-F	AATCCGAGATAGAGCGGTTC	1100	
ISAba2-R	TGACACATAACCTAGTGCAC		
ISAba3-F	CAATCAAATGTCCAACCTGC	403	
ISAba3-R	CGTTTACCCCAAACATAAGC		
IS18-F	CACCCAACTTTCTCAAGATG	925	
IS18-R	ACCAGCCATAACTTCACTCG		
CTX-F	GACGATGTCACTGGCTGAGC	500	[38]
CTX-R	AGCCGCCGACGCTAATACA		
SHV-F	AGGATTGACTGCCTTTTTG	392	[39]
SHV-R	ATTTGCTGATTTCGCTCG		
TEM-F	ATCAGCAATAAACCAGC	516	
TEM-R	CCCCGAAGAACGTTTTC		

**Table 2:** Antimicrobial resistance pattern of the isolates and the antibiotics to which each isolate was resistant.

Lab. ID	Year	Ward Isolation	Antimicrobial Resistance Pattern		
AB1	2019	WD	XDR	CRE	Am, Aug, Ptz, Kf, Ctt, Caz, Cro, Pime, Imp, Mer, Amk, Cp, Fd, Ts.
AB2	2019	OncW	MDR		Am, Aug, Ptz, Kf, Ctt, Caz, Cro, Pime, Imp, Mer, Amk, Fd.
AB5	2019	ER	SS		Am, Aug.
AB13	2019	ENT	SS		Am, Fd.
AB15	2019	OncW	MDR		Am, Cp, Ts.
AB18	2019	WD	XDR	CRE	Am, Aug, Ptz, Kf, Ctt, Caz, Cro, Pime, Imp, Mer, Amk, Gm, Cp, Fd, Ts.
AB21	2019	ER	XDR		Am, Aug, Ptz, Kf, Ctt, Caz, Cro, Pime, Imp, Mer, Amk, Gm, Cp, Fd, Ts.
AB31	2019	WD	XDR		Am, Aug, Ptz, Kf, Ctt, Caz, Cro, Pime, Imp, Mer, Amk, Gm, Cp, Fd, Ts.
AB52	2019	WD	XDR		Am, Aug, Ptz, Kf, Ctt, Caz, Cro, Pime, Imp, Mer, Cp, Fd, Ts.
AB64	2019	WD	XDR		Am, Aug, Ptz, Kf, Ctt, Caz, Cro, Pime, Imp, Mer, Amk, Gm, Cp, Fd, Ts.
AB69	2019	OW	XDR		Am, Aug, Ptz, Kf, Ctt, Caz, Cro, Pime, Imp, Mer, Amk, Gm, Cp, Fd, Ts.
AB85	2019	WD	MDR	MDR	Am, Aug, Kf, Ctt, Fd.
AB86	2019	OncW	XDR	CRE	Am, Aug, Ptz, Kf, Ctt, Caz, Cro, Pime, Imp, Mer, Amk, Cp, Fd, Ts
AB87	2019	WD	MDR	MDR	Am, Aug, Kf, Caz, Ctt, Fd, Fs.
AB76	2019	WD	XDR	CRE	Am, Aug, Ptz, Kf, Ctt, Caz, Cro, Pime, Imp, Mer, Amk, Gm, Cp, Fd, Ts.
AB93	2019	ICU	XDR		Am, Aug, Ptz, Kf, Ctt, Caz, Cro, Pime, Imp, Mer, Amk, Gm, Cp, Fd, Ts.
AB95	2019	Neuro	XDR		Am, Aug, Ptz, Kf, Ctt, Caz, Cro, Pime, Mer, Amk, Cp, Fd, Ts.
AB97	2019	ICU	XDR		Am, Aug, Ptz, Kf, Ctt, Caz, Cro, Pime, Imp, Mer, Amk, Gm, Cp, Tig, Fd, Ts.
AB59	2019	ICU	XDR		Am, Aug, Ptz, Kf, Ctt, Caz, Cro, Pime, Imp, Mer, Amk, Gm, Cp, Fd, Ts.
AB67	2019	WD	XDR		Am, Aug, Ptz, Kf, Ctt, Caz, Cro, Pime, Imp, Mer, Amk, Gm, Cp, Fd, Ts.
AB70	2019	WD	XDR		Am, Aug, Ptz, Kf, Ctt, Caz, Cro, Pime, Imp, Mer, Amk, Gm, Cp, Tig, Fd, Ts.
K17	2017	WD	MDR	ESBL ESBL	Ams, Ptz, Caz, Pime, Min.
K19	2017	WD	MDR		Ams, Ptz, Caz, Pime, Min, Levo, Ts.
K29	2017	ICU	MDR		Ams, Ptz, Caz, Pime, Azt, Gm, To, Cp, Levo, Ts, Min.
K32	2017	OPD	MDR		Ams, Ptz, Caz, Pime, Azt, Cp, Ts.
K34	2017	ICU	MDR		Ams, Ptz, Caz, Pime, Azt, Gm, To, Cp, Levo, Ts, Min.
K56	2017	WD	MDR		Ams, Ptz, Caz, Pime, Azt, Gm, To, Cp, Levo, Ts, Min.
K61	2018	ER	MDR		Ams, Ptz, Caz, Pime, Azt, Gm, To, Cp, Levo, Ts.
K69	2018	WD	MDR		Ams, Ptz, Caz, Pime, Azt, Cp, Levo, Ts, Min.
K72	2018	ICU	MDR		Caz, Pime, Azt, Cp, Levo, Ts.
K76	2018	OPD	MDR		Caz, Pime, Azt, Cp, Ts.
K79	2018	ICU	MDR		Kf, Caz, Cxm, Fd, Pime,
K89	2018	OPD	MDR		Ams, Ptz, Caz, Pime, Cxm, Fd, Ts, Aug, Cp.
K99	2018	ICU	MDR		Ams, Ptz, Caz, Pime, Cxm, Fd, Ts, Aug, Cp, Gm.
K109	2018	WD	MDR		Ams, Ptz, Caz, Pime, Cxm, Fd, Ts, Aug, Cp,
K112	2018	ICU	MDR		Ams, Ptz, Caz, Pime, Cxm, Fd, Ts, Aug, Cp,
K115	2018	ICU	MDR		Ams, Ptz, Caz, Pime, Cxm, Fd, Ts, Aug, Cp,
K118	2018	ICU	MDR		Ams, Ptz, Caz, Pime, Cxm, Fd, Aug, Cp.
K128	2018	ICU	MDR		Ams, Ptz, Caz, Pime, Cxm, Fd, Aug, Cp.
K132	2018	ICU	MDR		Ams, Ptz, Caz, Pime, Cxm, Fd, Aug, Cp, Ts, Gm.
K133	2018	WD	MDR		Ams, Ptz, Caz, Pime, Cxm, Fd, Aug, Cp, Ts, Gm.
K134	2018	WD	MDR		Ams, Ptz, Caz, Pime, Cxm, Fd, Aug, Cp, Ts, Gm.
K135	2018	WD	MDR		Ams, Ptz, Caz, Pime, Cxm, Fd, Aug, Cp, Ts, Gm.
K136	2018	ICU	MDR		Ams, Nor, Caz, Cp, Gm, Ts, Pime.
K137	2018	ICU	MDR		Ams, Ptz, Cxm, Caz, Pime, Cxm, Fd, Aug, Cp, Ts.
K139	2018	WD	MDR		Ams, Ptz, Cxm, Caz, Pime, Cxm, Fd, Aug, Cp, Ts, Gm.
K147	2018	ICU	MDR		Ams, Ptz, Caz, Pime, Azt, Gm, To, Cp, Levo, Min.
K148	2018	WD	MDR		Ams, Ptz, Caz, Pime, Azt, Gm, To, Cp, Levo, Min.
K9	2017	OPD	MDR		Ams, Ptz, Caz, Pime, Azt, Gm, To, Cp, Levo, Ts.
K10	2017	WD	MDR		Ams, Ptz, Caz, Pime, Azt, Gm, To, Cp, Levo, Min.
K14	2017	WD	MDR		Ams, Ptz, Caz, Pime, Min.
K1	2017	OPD	XDR		Ams, Ptz, Caz, Pime, Azt, To, Cp, Levo, Cst, Ts.

The year of collection, hospital ward, and isolate laboratory codes are shown. Carbapenem-resistant Enterobacteriaceae (CRE) resistant to one antibiotic in 3 antibiotic categories (MDR), extended-spectrum beta lactamase (ESBL), susceptible strain (SS), emergency room (ER), intensive care unit (ICU), OPD (outpatient department), ward (WD), oncology ward (OncW), neurology (Neu), ampicillin/sulbactam (AMS), amoxicillin/clavulanic acid (Aug), amoxicillin (Am), piperacillin/tazobactam (Ptz), ceftazidime (Caz), cefepime (Pime), cephalotin (Kf), cefoxitin (Ctt), ceftriaxone (Cro), cefuroxime (Cxm), aztreonam (Azt), ertapenem (Etp), imipenem (Imp), meropenem (Mer), amikacin (Amk), gentamicin (Gn), tobramycin (To), ciprofloxacin (Cp), minocycline (Min), norfloxacin (Nor), levofloxacin (Levo), nitrofurantoin (Fd), colistin (Cst), tigecycline (Tig), and trimethoprim/sulfamethoxazole (Ts).

**Table 3:** Genotype characteristics of 51 clinical isolates of *Klebsiella pneumoniae*.

Resistance-Determining Agents	Genotype	Number of Isolates (%)
OXA carbapenemases	OXA-23	34 (67)
Absence of OXA-23 and OXA-40		17 (33)
Metallo-beta-lactamase (MBLs)	SIM	22 (43)
	IMP	3 (6)
Co-existence of SIM and IMP		12 (23.5)
Absence of SIM and IMP		14 (27.5)
Overall carriage/absence of MBLs		37 (72.5)/14 (27.5)
Insertion sequences	IS*Aba1*	1 (2)
	IS*Aba2*	1 (2)
	IS*18*	11 (21.6)
Co-existence of IS*Aba1* and IS*Aba2*		1 (2)
Co-existence of IS*Aba1* and IS*18*		0 (0)
Co-existence of IS*Aba2* and IS*18*		20 (39)
Co-existence of IS*Aba1*, IS*Aba2,* and IS*18*		1 (2)
Absence of IS*Aba1*, IS*Aba2,* and IS*18*		16 (31.4)
Overall carriage/absence of *IS*		35 (69)/16 (31)
Extended-spectrum beta lactamase (ESBL)	TEM	2 (4)
	CTX	1 (2)
	SHV	2 (4)
Co-existence of TEM and SHV		5 (9.8)
Co-existence of TEM and CTX		4 (7.8)
Co-existence of SHV and CTX		22 (43)
Co-existence of TEM, SHV, and CTX		14 (27)
Absence of TEM, SHV, and CTX		1 (2)
Overall carriage/absence of ESBL		50 (98)/1 (2)

Two sample *t*-test between percentages were employed via the StatPac Version 4 statistics calculator to determine significance between percentages with groups. OXA-40 and ISAb3 were tested for but were not detected in all of the samples.

## Data Availability

Links for partial data are provided in the Supplementary Tables. Data are present in the paper, but the corresponding author can also be contacted should the need arise.

## Supplementary Materials

The following are available online at https://www.mdpi.com/article/10.3390/pathogens10091097/s1. Figure S1: Gel electrophoresis images of amplified resistance determinant genes by multiplex PCR of *Klebsiella pneumoniae* with the primers listed in Table 1. Table S1: Molecular genotyped links for 16S rRNA partial sequences with the GenBank ascension numbers deposited in NCBI databases.
