# Supplementary figures and images for: Genetic Analysis, Population Structure, and Characterisation of Multidrug-Resistant Klebsiella pneumoniae from the Al-Hofuf Region of Saudi Arabia
Source: Pathogens. 2021 Aug 28;10(9):1097. doi: 10.3390/pathogens10091097 (PMC8468815; doi:10.3390/pathogens10091097)

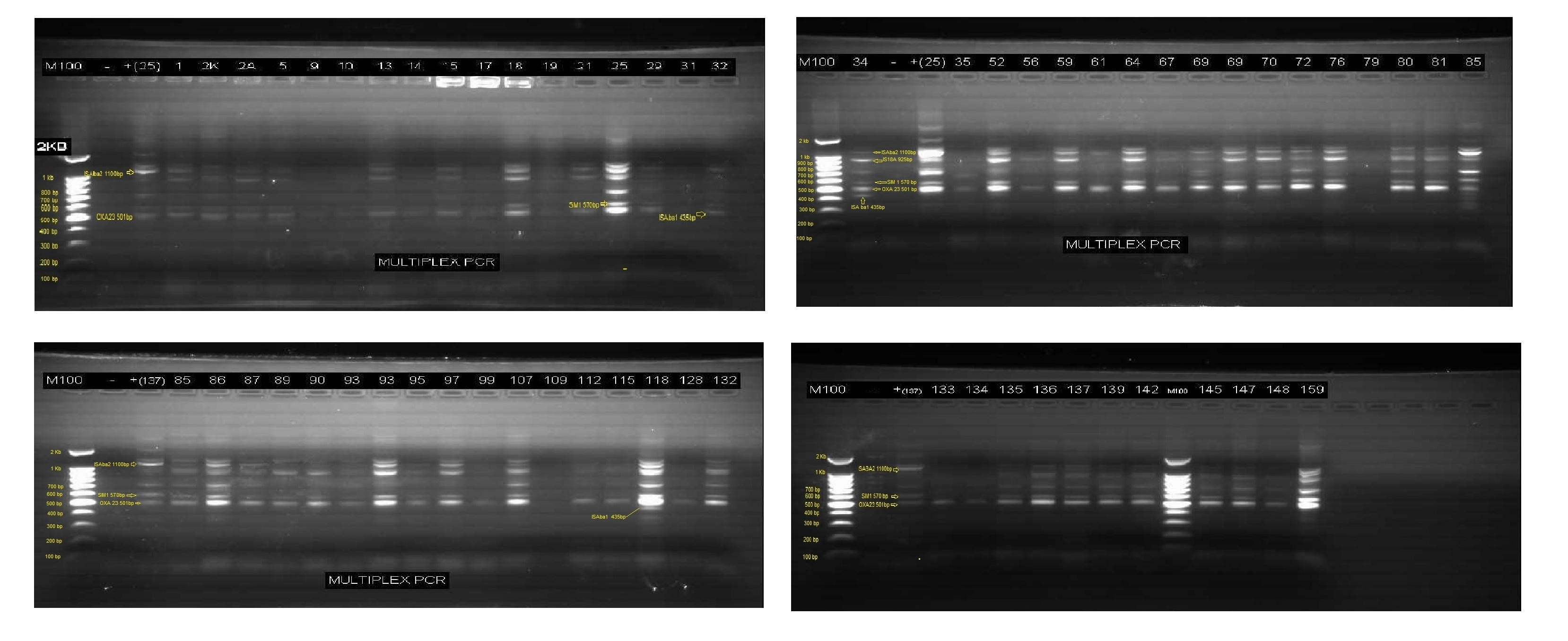

Supplement: Supplementary file 1 [file pathogens-10-01097-s001.zip › SS Figure 1 (2).jpg]
